# How does world economic policy uncertainty influence the carbon dioxide emission reporting and performance? Study of Fortune Global 500 firms

**DOI:** 10.1007/s11356-024-32643-0

**Published:** 2024-03-04

**Authors:** Antonios Persakis, Athanasios Fassas, Andreas Koutoupis

**Affiliations:** Department of Accounting and Finance of the School of Economic and Business Administration, University of Thessaly, Larissa-Trikala Ring Road, 415 00 Larissa, PC Greece

**Keywords:** World Economic Policy Uncertainty Index, Carbon dioxide emission reporting and performance, Institutional ownership, Industry affiliation, Fortune Global 500

## Abstract

This study contributes significantly to the field by utilising the World Economic Policy Uncertainty (WEPU) Index, as devised by (Ahir in Nat Bureau Econ Res 2022), to scrutinise its impact on carbon dioxide emission reporting and performance. Employing the generalised method of moments (GMM) on a substantial dataset of 604 Fortune Global 500 firms spanning from 2005 to 2020, our analysis reveals crucial insights. The research elucidates the dual influence of WEPU Index: a positive correlation with carbon dioxide emission reporting and a negative correlation aimed at mitigating adverse effects and promoting sustainable practices, thereby enhancing firm trust. Moreover, the findings shed light on how companies in emission-intensive industries tend to ramp up carbon dioxide emission reporting, potentially to bolster investor confidence, particularly during high WEPU Index periods. Furthermore, this study uncovers a compelling association between high emitters and lowered carbon dioxide emission performance, stemming from political and social pressures to integrate environmental considerations. Notably, this pressure intensifies during periods of increased WEPU Index. The empirical results presented in this study carry immediate practical implications. Specifically, they offer valuable insights for regulatory bodies and industry associations, guiding the development of enhanced environmental and social reporting regulations and guidelines, particularly concerning carbon emission reporting and performance.

## Introduction

Carbon dioxide (CO_2_) emissions are one of many ubiquitous contaminants in our environment that have been associated with deleterious effects on human health in various ways, such as inhalation problems (puffiness), resulting in even more carcinogenic effects through long-term exposure (Fernando and Hor 2017; Dong et al. 2018; Farooq et al. 2019), agriculture (Ashmore 2005) and climate change caused by rising global warming (Ramanathan and Carmichael 2008). Therefore, reducing carbon dioxide emissions is a necessary task for each country in order to address the severe challenges arising from environmental and human health degradation. Existing papers focus on the influencing factors of carbon dioxide emissions (e.g. Mutascu 2018; Liu et al. 2019; Jiang et al. 2019), but previous findings neglect macroeconomic and institutional factors that may link with carbon dioxide emissions. World uncertainty is one of these macroeconomic institutional factors that affects the external business environment of firms, which, in turn, affects their decision-making (Jiang et al. 2019; Yu et al. 2021). Furthermore, carbon dioxide emissions are related to the production decisions of firms (Song et al. 2017; Chen et al. 2019a; Wang et al. 2020). Therefore, this research enlightens the relationship between the World Uncertainty Index (WUI) developed by Ahir et al. (2022) and carbon dioxide emission reporting and performance using a large sample obtained from the Fortune Global 500.

As part of firms’ responsibility to mitigate events that raise global uncertainty (e.g. climate change, the global financial crisis of 2008, the sovereign debt crisis in Europe, the US fiscal cliff and sovereign debt crisis in Europe, the UK’s referendum vote in favour of BREXIT, US presidential elections, the coronavirus outbreak), firms are motivated and obliged to disclose information about carbon dioxide emissions they produce in order to ensure that any reported carbon dioxide emission disclosures are credible. Therefore, carbon dioxide emission disclosure can be a tool for companies to communicate environmental concerns and responsibilities to the public and stakeholders so that carbon dioxide emission disclosure can improve the firms’ image and sustainability (Hardiyansah et al. 2020). In addition, Simnett and Nugent (2007) claim that an important step in the direction of reducing carbon dioxide emissions is the disclosure by companies of reliable and relevant information regarding these emissions, facilitating emission trading schemes and aiding management and investor decision-making. Therefore, this study focuses on the effects of the WUI developed by Ahir et al. (2022) on carbon dioxide emission reporting and performance. Our results indicate that, although governments and regulators try to formulate regulatory changes to anticipate the consequences of events that raise global uncertainty, carbon dioxide emission reporting increases and carbon dioxide emission performance still deteriorates during periods of high policy uncertainty.

Subsequently, we investigate the impact of institutional ownership and industry affiliation that could potentially offset the impact of the WUI developed by Ahir et al. (2022) on carbon dioxide emission reporting and performance. In particular, different ownership structures imply different incentives to control and monitor a firm’s management (Shleifer and Vishny 1986; Morck et al. 1988). Therefore, concerning how ownership structure influences carbon emission disclosure, previous literature illustrated that it should communicate with institutional owners and minority controlling shareholders’ firms’ environmental achievements and then strengthen their investment confidence and improve shareholders’ interests (Solikhah et al. 2021). Furthermore, Liu et al. (2019) and Yu et al. (2021) present a relatively positive effect of ownership structure on corporate environmental management. In this regard, we add to the findings of previous literature by investigating the effects of institutional ownership on carbon dioxide emission reporting and performance under uncertainty. We find that institutional ownership is positively associated with carbon dioxide emission reporting. Furthermore, we illustrate that these results appear to be more prominent during periods of high policy–induced uncertainty.

In addition, according to Faisal et al. (2018) and Saraswati et al. (2021), carbon emission reporting is positively associated with high-profile industries. It means that firms in these industries have more incentives to provide environmental disclosures in order to maintain their legitimacy (Brammer and Pavelin 2006). Furthermore, Yuan et al. (2022) find that high-profile industries tend to decrease carbon dioxide emission performance. Therefore, we give further insights to previous literature investigating if carbon dioxide emission reporting and performance are influenced more by firms operating in emission-intensive industries. We show that there is a positive association between high-profile industries and carbon dioxide emission reporting and an inverse relationship between carbon dioxide emission performance and industry affiliation, whose effects are more pronounced in periods of high uncertainty.

Our paper makes the following contributions to the literature. First, although few papers have examined how periods of high policy–induced uncertainty influence carbon dioxide emissions, this study contributes to existing literature by giving insights into the fluctuations of carbon dioxide emission reporting and performance in periods of high world economic policy uncertainty developed by Ahir et al. (2022). Mainly, previous literature concentrates on examining the influence of subtypes of economic policy–induced uncertainty, like environmental uncertainty, on carbon dioxide emission reporting. For example, Persakis (2023) examines the relationship between environmental, social and governance (ESG) performance, firm performance, and carbon dioxide emission performance by utilising the Climate Policy Uncertainty Index (CPUI), an index that is developed to measure uncertainty about future government policies, international agreements and regulations aimed at mitigating climate change, reducing greenhouse gas emissions, promoting renewable energy or addressing environmental concerns. On the contrary, we use the world economic policy uncertainty developed by Ahir et al. (2022), which aims to measure the uncertainty stemming from changes in fiscal policies, monetary policies, trade regulations, geopolitical events or other economic policy–related decisions made by governments or international organisations. In this regard, we enhance the findings of Abu-Rahma and Jaleel (2019), Phan et al. (2020) and Persakis (2023) investigating the influence of economic policy uncertainty on carbon dioxide emission disclosure and performance. Our study contributes to the extant body of literature by presenting empirical evidence in underexplored domains regarding the impact of interactions among the recently introduced new World Economic Policy Uncertainty Index developed by Ahir et al. (2022), institutional ownership and industry affiliation on carbon dioxide emission reporting and performance. Specifically, our findings indicate that (a) institutional ownership exhibits a positive association with carbon dioxide emission reporting, even in the presence of elevated uncertainty, and (b) industries with a prominent public profile tend to exhibit higher levels of carbon dioxide emission reporting. Third, the utilisation of a sample encompassing multiple years and industries enhances the validity of this contribution to the realm of research in international accounting and finance. Hence, the utilisation of an extensive sample derived from the Fortune Global 500 list spanning the fiscal years 2005–2020 will augment the global scope of the research outcomes. The significance of utilising this list resides in the observation that the aggregate revenues generated by the companies included therein amount to over 50% of the total global revenues of the listed companies on a worldwide scale. Fourth, the inclusion of the WUI, as devised by Ahir et al. (2022), bolsters our research findings. This index has been deemed superior to alternative measures of uncertainty, as highlighted by Gozgor et al. (2019), due to its unique ability to construct an uncertainty index specifically tailored for a panel dataset encompassing both developed and developing nations. In essence, it exhibits comparability among nations. Fifth, by applying a generalised method of moments (GMM) framework, we are able to (a) control for firm-specific effects, (b) deal with the inclusion of the lagged dependent variable as an explanatory variable and (c) control for the problem of endogeneity of the regression predictors (Staikouras and Wood 2004). Sixth, the study enhances the robustness of its findings by incorporating alternative indicators for carbon emission reporting, including the WUI and ownership structure. This practice ensures the reliability and consistency of the findings, enhancing the overall credibility of the study’s conclusions.

The rest of this paper is managed as follows. Section ‘Literature review and hypothesis development’ shows the literature review and the research hypotheses. Section ‘Sample selection and methodology’ introduces the empirical model and data. Section ‘Empirical results’ presents the empirical results and discussion. Section ‘Robustness tests’ implements robustness tests, and section ‘Conclusions and policy implications’ provides the conclusion.

## Literature review and hypothesis development

### World economic policy uncertainty and carbon dioxide emission reporting and performance

Carbon dioxide emission reporting and performance as a climate change management tool play a significant role in modern stakeholder relations (Velte et al. 2020). Carbon dioxide emission reporting discloses historic and prospective carbon dioxide emission performance to investors, as well as other climate-related information (Pitrakkos and Maroun 2020). Carbon dioxide emission performance is the outcome of managerial activity that contributes to carbon dioxide emissions (Busch and Lewandowski 2018).

To our knowledge, there is no previous evidence of how the World Economic Policy Uncertainty Index developed by Ahir et al. (2022) influences carbon dioxide reporting. However, there is scant evidence exploring the effects of subtypes of policy-induced uncertainty, like environmental uncertainty, on carbon dioxide emission reporting. Lin and Ho (2011) conclude that environmental uncertainty has a negative and significant impact on green practice adoption. Pondeville et al. (2013) claim that environmental uncertainty is an unpredictable situation, for example natural disasters or climate change or the rate of market change, for instance competitor challenges, technological changes and customer desires that can result in companies being better able to adapt well in the present and the future. They show that firms with high ecological, environmental uncertainty would result in the underdevelopment of a proactive environmental strategy, environmental information system or formal environmental management control system. Abu-Rahma and Jaleel (2019) reveal that environmental uncertainty affects business in an organisation, affecting environmental information in decision-making. Another strand of literature shows that uncertainty has a positive effect on firm’s environmental disclosure by incorporating difference-in-difference estimation (Phan et al. 2020). In detail, they point that firms have incentives to reduce the transparency of carbon dioxide emission information disclosure when the uncertainty is at a high level.

In addition, quite a few studies explore the impact of macroeconomic factors such as the presence of an emission trading scheme, the level of economic development, the legal system, regulatory governance and national culture on carbon dioxide emission reporting (Akhiroh and Kiswanto 2016; Kilic and Kuzey 2019; He et al. 2019). At the microeconomic level, there are various papers that examine the influence of institutional ownership, corporate governance, firm size and firm performance on carbon emission disclosure (Akhiroh and Kiswanto 2016; Kalu et al. 2016; Kilic and Kuzey 2019; Hermawan et al. 2018; Alfani and Diyanty 2020). Information asymmetry is another factor that directly influences corporate carbon emissions. Hahn et al. (2015) provide evidence that information disclosure reduces information asymmetries between a firm and its potential shareholders, which, in turn, lowers transaction costs. In addition, Kothari et al. (2009) suggest that disclosing information creates uncertainties, which, in turn, enhance information asymmetries. Therefore, regarding the effects of information asymmetry on carbon dioxide emissions, previous evidence shows that reporting a higher exposure to physical risks is associated with lower information asymmetry for firms falling under the regulation of the EU Emissions Trading Scheme (Schiemann and Sakhel 2019). Another strand of literature investigates the inverse effect of carbon emission disclosure on information asymmetry. Borghei et al. (2018) and Adhikari and Zhou (2021) show that voluntary carbon disclosure is negatively related to information asymmetry. In detail, they illustrated that firms choose to disclose carbon emission information in order to reduce information asymmetry between management and investors, allocating scarce resources efficiently.

In periods of high levels of world policy–induced uncertainty, countries feature higher information asymmetries (Ha et al. 2021). Furthermore, Nagar et al. (2019) find that economic policy uncertainty is associated with increased bid-ask spreads, and managers respond to this uncertainty by increasing their voluntary disclosures to mitigate this bid-ask spread increase. Jin et al. (2019) state that if economic policy uncertainty affects crash risk, the managers of firms with higher information asymmetry are more likely to conceal bad news because it is harder for investors to obtain information from firms with higher information asymmetry in an uncertain environment.

According to the above analysis, information asymmetry increases in an uncertain environment, and carbon emission disclosure increases in periods of high information asymmetry in order to minimise the negative effects of these information asymmetries. Therefore, this paper enhances the results of previous literature (e.g. Abu-Rahma and Jaleel 2019; Pan et al. 2020) by providing evidence of how important events related to uncertainty worldwide influence carbon dioxide emission reporting. Based on the aforementioned analysis, it can be inferred that information asymmetry tends to escalate in situations characterised by uncertainty. Consequently, in order to mitigate the adverse consequences arising from such information asymmetries, there is a propensity for an upsurge in carbon emission disclosure during periods marked by heightened levels of information asymmetry. Hence, this study contributes to the existing body of literature (e.g. Abu-Rahma and Jaleel 2019; Pan et al. 2020) by presenting empirical evidence on the impact of significant global uncertainties on carbon dioxide emission reporting. Within the theoretical framework of economics (Becker 2017), the correlation between world economic policy uncertainty and the reporting of carbon dioxide emissions can be analysed by examining how economic policy uncertainty influences the actions of businesses operating in the international market. The decision-making and behaviour of corporations are directly influenced by economic policies (Jackson and Orr 2019; Chin et al. 2021). The presence of ambiguity in policies pertaining to trade, taxation or environmental regulations can exert a substantial impact on the decision-making processes of businesses, affecting their investment choices, operational strategies and reporting practices (Bloom et al. 2007; Gulen and Ion 2016). In the presence of economic policy uncertainty, firms may modify their disclosure practices regarding carbon emissions as a reaction to perceived alterations in regulatory frameworks, financial vulnerabilities and market dynamics. The presence of uncertainty in economic policies may result in modifications to reporting accuracy, transparency and the overall prioritisation of environmental metrics in financial disclosures. In response to regulatory changes or stakeholder perceptions, certain companies may opt to disclose additional information as a means to mitigate potential risks. Conversely, other companies may opt to reduce transparency in order to navigate competitive environments during periods of economic uncertainty. Therefore, under economic theory, it is expected that, under high-level economic policy uncertainty, firms will increase information related to carbon emissions in order to minimise uncertainty and enhance firm trust. Thus, we forward the following first hypothesis:

- **Hypothesis 1.** Word economic policy uncertainty has a positive impact on carbon dioxide emission reporting.

Accounting researchers debate the relationship between carbon dioxide emission reporting and performance. Hughes et al. (2001) reveal that differently rated companies follow different disclosure strategies and that those firms with the worst environmental performance disclose the most. Freedman and Jaggi (2011) suggest that higher emitters are likely to disclose more emission information. Sutantoputra et al. (2012) report that there is no significant relationship between carbon dioxide emission reporting and performance. Luo and Tang (2014) show that better environmental performers are associated with higher levels of carbon disclosures. Qian and Schaltegger (2017) state that the change in carbon disclosure levels is positively associated with carbon performance, which confirms that carbon disclosure motivates firms and creates an ‘outside-in’-driven effect for subsequent change and improvement in carbon performance. Luo (2019) gives evidence that there is a negative relationship between carbon disclosure and carbon emission performance, which is consistent with the legitimacy theory that carbon disclosure may be undertaken for the purposes of legitimation.

As legitimacy theory postulates that environmental disclosure is a function of pressure by investors, firms may disclose information to manipulate investors in order to gain their support and approval because it is often easier to manage a firm’s image than to make commitments to sustainability performance (Lyon and Maxwell 2011). Based on legitimacy theory and the results of Cho and Patten (2007), there is a negative association between environmental disclosure and performance. It means that worse environmental performers are found to make relatively more extensive disclosures to maintain their environmental reputation. Reversely, supporters of legitimacy theory do not presume the idea that disclosure will either reflect or affect performance. However, there is evidence that if environmental disclosure mitigates the effects of environmental performance on a firm’s reputation (Patten 2015), it will reduce the incentives for firms to manage environmental practices and ameliorate environmental performance.

Although there is no previous study that examines the effect of world-induced economic policy uncertainty developed by Ahir et al. (2022) on carbon emission dioxide performance, in recent years, there has been a scant and controversial literature examining how economic policy uncertainty, as a subtype of world-induced policy uncertainty, influences carbon dioxide emissions (e.g. Cai et al. 2018; Zhou et al. 2018; Dietz and Venmans 2019; Hebum et al. 2019; Jiang et al. 2019; Huang et al. 2020; Pirgaip 2020; Yu et al. 2021; Wei et al. 2022). Hepburn et al. (2019), Adedoyin and Zakari (2020) and Huang et al. (2020) show that economic policy does not affect emissions. Zhou et al. (2018) find that considering the effect of quality uncertainty on carbon emissions can effectively increase profit and reduce total carbon emissions for firms. Jiang et al. (2019) indicate that carbon dioxide emissions are affected by economic policy uncertainty when the growth of carbon emissions is in a higher or lower growth period. Dietz and Venmans (2019), Pirgaip and Dincergok (2020) and Yu et al. (2021) show that economic policy uncertainty imposes a significantly positive impact on firms’ carbon emission intensity because of the increase in energy consumption using coal and fossil energy. Wei et al. (2022) find that an increase in international crude oil price uncertainty can inhibit the company’s carbon emission due to a shift in the use of energy from oil to renewable energy sources.

In addition, few papers examine the fluctuations in carbon dioxide emission intensity during different levels of the business cycle. However, there is no consensus about this relationship. Alege et al. (2017) note that carbon emissions are typically pro-cyclical; they increase during expansions and decrease during recessions, and in some cases, they are countercyclical; they reduce during periods of economic expansion or growth. Shahiduzzaman and Layton (2015) and Blazquez et al. (2017) find that aggregate emissions and emission intensity reduce much faster in contractions than they increase in expansions. On the contrary, Khan et al. (2019) and Skare et al. (2021) suggest that carbon emissions are moving together with economic shocks (high synchronicity), particularly at troughs and peaks of a business cycle. In addition, the study conducted by Sarwar et al. (2017) emphasise the correlation between renewable consumption of electricity and economic growth, as well as the inverse correlation between oil prices and economic growth. They also suggest that these relationships have the potential to have a detrimental impact on carbon emission intensity. According to Sarwar et al. (2017), there exists a positive correlation between economic growth and education. Additionally, it has been discovered that there is a noteworthy and adverse correlation between education and greenhouse gas emissions. This implies that by enhancing educational standards, it is possible to mitigate greenhouse gas emissions.

In respect to the aforementioned papers, there is ambiguity about how changes in carbon emission performance may be influenced by disclosure changes. Following the legitimacy theory, it is expected that there is no or little negative influence of carbon emission disclosure on carbon emission performance, given that increasing disclosures is viewed as an approach to enhancing sustainable images (Milne et al. 2009). Furthermore, there is no consensus among the results of previous literature examining the change in carbon emission performance under periods of uncertainty. Previous literature mainly examined how economic growth and energy consumption are influenced in periods of economic growth and recession and how these effects are related to carbon emission intensity. Cowan et al. (2014) and Chen et al. (2019b) indicate that there are mixed effects of the business cycle on energy consumption and, therefore, on carbon emission performance. In this case, this paper aspires to enhance previous literature investigating how important events related to world economic policy uncertainty influence carbon dioxide emission performance. It is expected that pressures to increase environmental disclosure quality during periods of uncertainty could improve social and environmental performance (Schaltegger and Csutora 2012), which would lure managers to follow valuable and constructive changes in strategic thinking and convert data into action (Topping 2012; Vesty et al. 2015), leading to lower carbon dioxide emissions. The relationship between world economic policy uncertainty and carbon dioxide emission performance can be analysed by applying the resource-based view (RBV) theory. In the context of this theoretical framework, companies that encounter uncertain economic policies frequently engage in resource reallocation, innovation and strategic decision-making as a means to effectively navigate regulatory environments (Hart and Dowell 2011). The presence of uncertainty may incentivise firms to allocate resources towards the development and implementation of environmentally friendly technologies, as well as to adjust their operations in response to evolving regulatory frameworks. Consequently, this proactive approach has the potential to result in a reduction in their overall carbon emissions. According to the RBV framework, organisations that possess the capacity to efficiently manage their resources and navigate unpredictable policies have the potential to achieve a competitive advantage through the cultivation of innovation, collaboration and the establishment of sustainable practices. Therefore, under the RBV theory, the following second hypothesis is proposed:

- **Hypothesis 2.** Word economic policy uncertainty has a negative impact on carbon dioxide emission performance.

### The moderating role of institutional ownership

Extant literature suggests that different ownership structures imply different incentives to control and monitor a firm’s management (Shleifer and Vishny 1986; Morck et al. 1988). Therefore, according to Jensen and Meckling (1976), monitoring managerial decisions becomes essential to assure that shareholders’ interests are protected and to ensure reliable and complete financial reporting. Many previous empirical studies investigated the association between ownership structure and corporate disclosure, and their findings are contradictory (Abu Qa’dan and Suwaidan 2019). For example, institutional ownership encourages more optimal management supervision to impede opportunistic manager behaviour (Widyaningsih et al. 2017). Therefore, there is a direct positive and significant correlation between institutional ownership and corporate disclosure (Lin et al. 2018; Nurleni et al. 2018). Previous literature shows that there is a mixed association between ownership concentration and corporate disclosure. It also confirms that a higher level of ownership concentration may provide less information disclosure because shareholders have internal authority for obtaining information, and therefore, there is a negative association between management ownership concentration and the level of corporate disclosure (Pongsaporamat 2020). However, Jiang et al. (2011) demonstrate that ownership concentration and level of disclosure have no significant relationship. On the contrary, Khan et al. (2013) and Laksmi and Kamila (2018) demonstrate that state ownership has significant positive effects on the disclosure of corporate social responsibility.

Especially regarding the effect of ownership structure on carbon dioxide emission reporting, previous literature illustrates that corporations with greater ownership concentration should disclose more environmental information, communicate with institutional owners and minority controlling shareholders’ environmental achievements, and then strengthen their investment confidence and improve shareholders’ interests (Hermawan et al. 2018; Solikhah et al. 2021). In contrast to the results of Ho and Tower (2011), Cotter and Najah (2012) and Chang and Zhang (2015), who find that firms with greater foreign and institutional ownership have a significant and positive correlation with climate change disclosures, Akhiroh and Kiswanto (2016) and Hermawan et al. (2018) suggest that foreign and institutional ownership does not affect carbon emission disclosure. On the contrary, Alhazaimeh et al. (2014) find that there is a significant and negative relationship between institutional ownership and voluntary disclosure. Kim et al. (2021) state that foreign investors can suggest that management hires a sufficient number of outside directors to enhance the firms’ transparent accounting environment. Therefore, they find that foreign investors are likely to demand voluntary reporting of carbon emissions. Solikhah et al. (2021) indicate that institutional ownership has a positive effect on the disclosure of carbon emissions, while environmental performance and managerial ownership do not affect the reporting of carbon emissions.

In a similar way, carbon dioxide emission performance is another new area that may be influenced by ownership structure. Earnhart and Lizal (2006) conclude that increased state ownership improves environmental performance relative to all other ownership types, even though the state is more likely to have retained ownership in high-polluting industries. Wang and Jin (2007) show that the community owned in China has better environmental performance in water pollution discharges than the state-owned and privately owned, suggesting that the community-owned may internalise environmental externalities. Walls et al. (2012) and Kock and Min (2016) indicate that firm governance mode, e.g. ownership structure, is linked with environmental management. Calza et al. (2016) illustrate that ownership structure matters for firms’ environmental proactivity. Specifically, they show that firms with a higher percentage of state ownership present superior green proactivity, while ownership concentration appears negatively related to proactive environmental strategy. Liu et al. (2019) present a relatively positive effect of ownership structure on corporate environmental management. Yu et al. (2021) find that family firms with ownership, operational and strategic control can achieve higher environmental performance within a province with more stringent environmental regulations.

Regarding the change in ownership structure during periods of uncertainty, there is scarce evidence. Filatotchev and Nakajima (2010) argue that ownership structure is an important dimension of corporate governance that has a prominent effect on firm performance. According to Akhiroh and Kiswanto (2016), the greater the institutional ownership, the greater the institutional encouragement to supervise the management of the firm so as to optimise firm performance. In this regard, Doan et al. (2020) state that increased economic uncertainty is associated with lower performance levels for state-owned firms, whereas foreign-owned firms can better mitigate the negative impact of their performance than domestic-owned firms. Furthermore, Devos et al. (2013) find that institutional ownership increased prior to the financial crisis, declined significantly during the period of market stress, but rebounded after.

- **Hypothesis 3.** Institutional ownership has a significant positive effect on the carbon dioxide emission reporting.
- **Hypothesis 3a.** An increase of word economic policy uncertainty is associated with higher carbon dioxide emission reporting for institutional-owned firms.
- **Hypothesis 4.** Institutional ownership has a significant negative effect on the carbon dioxide emission performance.
- **Hypothesis 4a.** An increase of word economic policy uncertainty is associated with lower carbon dioxide emission performance for institutional-owned firms.

### The moderating role of industry affiliation

Firms are more likely to disclose information in accordance with the characteristics of their industry. The industry is considered a proxy for legitimacy theory in a number of greenhouse gas emission disclosure research papers. That is, firms in highly polluting industries are more likely to disclose carbon information publicly (Faisal et al. 2018; Saraswati et al. 2021). Luo et al. (2012), Choi et al. (2013) and Faisal et al. (2018) note that firms in environmentally visible industries have strong incentives to proactively and promptly react to social and political pressure and provide more voluntary environmental disclosures in order to maintain their legitimacy. Liu and Anbumozhi (2009) demonstrate the positive impact of environmentally sensitive industries on environmental disclosure. Prado‐Lorenzo et al. (2009) show that all industries (e.g. airlines, chemicals, energy, forest and product papers, industrial and farm equipment, metals, mining, motor vehicles and parts, petroleum refining, and utilities) except for the aerospace and defence industry are positively associated with environmental reporting practices. Rankin et al. (2011) find that firms belonging to the mining and energy sectors provide more credible and consistent greenhouse gas emission information. Matsumura et al. (2014) find that if greenhouse gas emission disclosure increases among peer companies in an industry, a company is more likely to release the same information. Saraswati et al. (2021) indicate that the industry type classified as high-profile has a positive effect on carbon emissions disclosures. In other words, they claimed that the higher the profile, the wider the disclosure will be, due to pressure from stakeholders.

Regarding the effects of industry on carbon dioxide emission performance, previous literature is limited (e.g. Tian et al. 2014; Dong et al. 2018). Tian et al. (2014) and Dong et al. (2018) illustrate that the secondary industry is energy-intensive, accelerating carbon emissions and carbon intensity. Specifically, Tian et al. (2014) claim that industrial structure changes involving a shift from agriculture, mining and light manufacturing to resource-related heavy manufacturing in many regions led to a rapid increase in carbon dioxide emissions at the national level. Furthermore, the tertiary industry is regarded as an industry with less energy consumption and thus lower emissions and carbon intensity. Liu et al. (2015) find that chemical, iron and steel, metal and machinery, and cement and ceramic subsectors contribute the most to the decline in energy intensity. In contrast, they find that the impacts from pulp and paper, other industries, fuel processing, and textile subsectors on the change in energy intensity are relatively minor.

In addition, previous literature gives evidence that positive and negative corporate and social responsibility news have significant impacts on the stock market value of firms in diverse industries (Pérez et al. 2020). However, they also do not confirm that all firms in high-profile industries accumulate a stronger impact of corporate and social responsibility news on the abnormal returns in the stock market when compared to low-profile industries. Pérez et al. (2020) show that although high-profile industries suffer from strong social pressures derived from their visibility, political risk and intense competition, investors increase their pressure on the firms that operate in high-profile industries to incorporate social and environmental considerations into their business activities. In the same vein, Solikhah (2016) suggests that high-profile industries disclose corporate and social reporting broader related to carbon emissions than low-profile industries because they have a high sensitivity to social and environmental problems that may arise. In periods of uncertainty, firms enhance corporate social responsibility in order to strengthen stakeholder trust and increase firm value (Rjiba et al. 2020). Ongsakul et al. (2021) demonstrate that in times of economic uncertainty, firms with larger managerial ownership invest more in corporate social responsibility, which offers insurance-like protection against these adverse events. In the same vein, Yuan et al. (2022) give support to previous evidence that firms tend to adopt more corporate social responsibility engagement during periods of higher uncertainty, as it is a positive signal to their stakeholders. These effects are more prominent for firms in more sensitive industries that are characterised by high sensitivity to the environmental and social environment, which further validates the sending signal mechanism.

In line with the aforementioned papers, we enhance previous literature by examining how the high- and low-profile^1^ industries affect carbon dioxide emission reporting and performance. We expect that carbon dioxide emission reporting will be greater for firms operating in emission-intensive industries in order to enhance investor trust in the quality of the information presented. On the contrary, it is expected that high-profile industries will lower carbon dioxide emissions due to enhanced corporate social responsibility in order to protect and strengthen customer loyalty and promote brand image. Furthermore, due to the lack of previous evidence about how high- and low-profile industries are influenced in periods of uncertainty, it is expected that the impact of WUI will be more prominent on the high-profile industry-carbon emission reporting and performance nexus because firms that belong to high-profile industries are characterised by high sensitivity to the environmental and social environment (Ongsakul et al. 2021). Therefore, we formulate the following fifth and sixth hypotheses with their subhypotheses:

- **Hypothesis 5:** Higher-profile industries are positively associated with carbon dioxide emission reporting.
- **Hypothesis 5a:** Compared with low-profile industry, an increase of word economic policy uncertainty is associated with higher carbon dioxide emission reporting for high-profile industry.
- **Hypothesis 6:** Higher-profile industries are negatively associated with carbon dioxide emission performance.
- **Hypothesis 6b:** Compared with low-profile industry, an increase of word economic policy uncertainty is associated with lower carbon dioxide emission performance for high-profile industry.

## Sample selection and methodology

### Sample selection

**Fig. 1:**
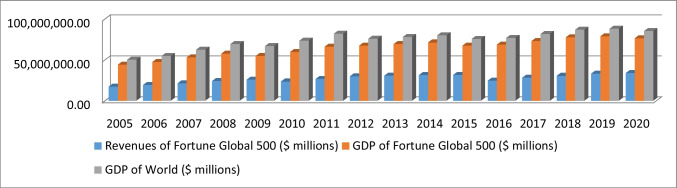
Revenues of Fortune Global 500 (in millions of dollars)

This study uses data from the Fortune Global 500 list for the fiscal years 2005–2020 that is compiled and published by Fortune magazine. The Fortune Global 500 is a ranking of the top 500 public firms worldwide as measured by revenue. The value of using this list lies in the fact that Fortune Global 500 firms generate revenues of more than one-third of the world’s GDP each examining year (see Fig. 1). Furthermore, the originality and uniqueness of using this population set are based on two characteristics: it comprises the market’s best performers and is geographically dispersed (Malik and Makhdoom 2016). Sledge (2011) states that this list is a good barometer of successful corporations around the world since it has tracked the numerous changes that have occurred in the global business landscape over the last 30 years. The initial sample consisted of 897 firms (8000 firm-year observations).

Based on several selection criteria, a part of the sample has to be excluded. First, firms within the finance sector, such as banks, insurance companies, real estate companies and other financial institutions, were excluded from the sample because it has been acknowledged that companies within this sector possess markedly different financial characteristics from other industry classifications. Therefore, the sample is further reduced to 685 firms (6059 firm-year observations).

Second, due to missing values, the final sample held 604 firms (5157 firm-year observations).

To avoid outlier concerns, the dependent, independent and control variables were winsorized at the 1st and 99th percentiles.

All data for examining firms is collected from DataStream, I/B/E/S, the official site of Yahoo Finance, and its corporate reports (annual, social responsibility and sustainability reports). The macroeconomic and other country institutional data are collected from stock market exchanges, the OECD, the Global Economy data, CEIC data and the World Bank data.

Table 1 presents an overview of the final sample’s distribution, categorised by industry in panel A, by regions in panel B and by year in panel C. The high-profile industries have the largest proportion (65.07%) of firms included in the Fortune Global 500 list for the fiscal years 2005–2020. Overall, Asia Pacific and the Americas regions have the largest proportion (35.43% and 34.27%, respectively) of firms in the Fortune Global 500 list for the fiscal years 2005–2020. However, there are no major differences in sample distribution by year.

**Table 1:** Sample distribution

	Frequency	Percent
Panel A: distribution by industry affiliation		
Industries		
1. High-profile industries	393	65.07
2. Low-profile industries	211	44.05
Total firms	*604*	
Panel B: distribution by regions		
Country		
1. Americas (Brazil, Canada, Chile, Colombia, Mexico, USA)	207	34.27
2. Asia Pacific (Australia, China, Hong Kong, India, Indonesia, Japan, Singapore, South Korea, Taiwan)	214	35.43
3. Europe (Austria, Belgium, Denmark, Finland, France, Germany, Hungary, Ireland, Italy, Netherlands, Norway, Poland, Portugal, Russia, Spain, Sweden, Switzerland, UK)	179	29.64
4. Middle East/Africa (Saudi Arabia, United Arab Emirates)	4	0.66
Total firms	*604*	
Panel C: distribution by year		
Year		
1. 2005	246	4.77
2. 2006	262	5.08
3. 2007	289	5.60
4. 2008	281	5.45
5. 2009	302	5.86
6. 2010	323	6.26
7. 2011	349	6.77
8. 2012	337	6.53
9. 2013	349	6.77
10. 2014	347	6.73
11. 2015	333	6.46
12. 2016	342	6.63
13. 2017	367	7.12
14. 2018	329	6.38
15. 2019	339	6.57
16. 2020	362	7.02
Total firm-year observations	*5157*	

### Variable selection

#### Carbon dioxide emission reporting measure

We apply the quality of carbon dioxide emission reporting developed by Pitrakkos and Maroun (2020) as a metric of carbon dioxide emission reporting (CER_*it*_) because it is the first measure that takes into account both the quality and quantity of disclosures dealing with greenhouse gas emissions among firms with a relatively large or small carbon footprint, as well as considering which disclosure media firms use (integrated vs. sustainability report) (Pitrakkos and Maroun 2020). It also provides a more nuanced perspective on how carbon emission disclosures are being used to manage stakeholders’ reporting expectations (Pitrakkos and Maroun 2020). Corporate carbon disclosure is identified through content analysis of each company’s annual reports. Freedman and Jaggi (2011) use content analysis as their research tool for measuring the disclosure of information about carbon dioxide emissions.

The quality of carbon dioxide emission reporting is defined in terms of eight characteristics/indicators, which suggest that the disclosures are more useful for assessing how a company is managing its greenhouse gas emissions (Pitrakkos and Maroun 2020). These include density (Density index_*it*_), attribute (Attribute index_*it*_), management orientation (Management orientation index_*it*_), integration (Integration index_*it*_), assurance (Assurance index_*it*_), strategy (Strategy index_*it*_), readability (Readability index_*it*_) and repetition (Repetition index_*it*_) indices. Each is used to construct an ordinal quality score for carbon reporting. Therefore, to assess the quality of carbon reporting, these eight indices are combined in Eq. (1), which is discussed in detail as follows. A higher value of the quality of carbon dioxide emission reporting index (CER_*it*_) suggests that firms assess more quantitative information on carbon emissions and are inclined to identify climate change as a strategic concern.

1 $${\text{CER}}_{it}={\text{ Density index}}_{it}+{\text{Attribute index}}_{it}+{\text{Management orientation index}}_{it}+{\text{Integration index}}_{it}+{\text{Assurance index}}_{it}+{\text{Strategy index}}_{it}+{\text{Readability index}}_{it}-{\text{Repetition index}}_{it}$$

where Density index_*it*_ is constructed and used to measure the level of detail being provided in an integrated report (Hrasky 2011; Michelon et al. 2015). Based on Pitrakkos and Maroun (2020), the index is the ratio between the total number of disclosures per data instrument (see Table 2) and the total number of pages in a report. A high-density index indicates a more concise report. This index has a minimum value of 0 (implying low quality) and an unlimited maximum value (indicating high quality). It is formulated as follows:

2 $${\mathrm{Density\, index}}_{it}= \frac{1}{{k}_{i}}\sum\nolimits_{j=1}^{{k}_{i}}{{\text{CD}}}_{ij}$$

where *k*_*i*_ is the number of pages found within the report of firm *i* and CD_*ij*_ is 1 where carbon disclosure item *j* for firm *i* can be categorised as per the data checklist. CD_*ij*_ is, otherwise, 0 (Pitrakkos and Maroun 2020).

**Table 2:** Disclosure themes

A. Emissions	This includes disclosures relating to an entity’s carbon emissions and how such emissions were calculated
B. Greenhouse gas emission intensity	This includes disclosures relating to an entity’s GHG intensity ratios and the variables which make up such ratios
C. Reduction of greenhouse gas emissions	This includes the reduction of an entity’s GHG emissions in totality or at an individual scope level
D. Assurance	This includes a disclosure relating the assurance of an entity’s GHG emissions
E. Actions	This includes the disclosure of either serious or general actions taken to reduce GHG emissions
F. Energy	This includes the disclosures of an entity’s renewable fuels, non-renewable fuels or energy reduction
G. Integration	This includes any disclosures relating to a company’s carbon footprint in relation to a company’s risk assessment and/or strategy
H. Targets	This includes any target set or achieved by a company in relation to its carbon emissions or energy consumption

Pitrakkos and Maroun (2020)

Attribute index_*it*_ is constructed and used to measure the type of information being reported (Michelon et al. 2015). According to Pitrakkos and Maroun (2020), the index is based on whether carbon disclosure (see Table 2) is qualitative or not. A higher-density index implies more quantitative and monetary information and higher-quality reporting (Michelon et al. 2015; McNally et al. 2017). The index has a minimum value of 0.33 (implying low quality) and a maximum value of 1 (indicating high quality). It is formulated as follows:

3 $${\mathrm{Attribute\, index}}_{it}= \frac{1}{{3n}_{i}}\sum_{j=1}^{{k}_{i}}{(w\times {\text{CD}}}_{ij})$$

where *n*_*i*_ is the total number of carbon disclosures found within the report of firm *i* and CD_*ij*_ is 1 where carbon disclosure item *j* for firm *i* can be categorised as per the data checklist. CD_*ij*_ is, otherwise, 0. *w* is 1 if the disclosed items contained qualitative information, 2 if the disclosed items contained quantified information and 3 if the disclosed item contained monetary information (Pitrakkos and Maroun 2020).

Management orientation index_*it*_ is constructed and used to measure whether a company has taken a symbolic or substantive approach to its carbon footprint (Michelon et al. 2015; Borghei et al. 2018). Each disclosure theme (see Table 2) is coded as rhetorical or committed. The index scores committed disclosures as 1 and rhetoric disclosures as 0 (Michelon et al. 2015). A higher management orientation index indicates that a report consists primarily of committed disclosures (implying higher quality). The index has a minimum value of 0 (implying low quality) and a maximum value of 1 (indicating high quality), and based on (Pitrakkos and Maroun 2020), it is formulated as follows:

4 $${\mathrm{Management\, orientation\,index}}_{it}= \frac{1}{{n}_{i}}\sum\nolimits_{j=1}^{{k}_{i}}{(m\times {\text{CD}}}_{ij})$$

where *n*_*i*_ is the total number of carbon disclosures found within the report of firm *i* and CD_*ij*_ is 1 where carbon disclosure item j for firm i can be categorised as per the data checklist. CD_*ij*_ is, otherwise, 0. *m* is 0 if the disclosed item contained rhetorical information and 1 if the disclosed item contained committed information (Pitrakkos and Maroun 2020).

Strategy index_*it*_ is constructed and used to measure how an organisation tailors its business model and strategy to respond to its external environment and the risks and opportunities it faces (Pitrakkos and Maroun 2020). According to Pitrakkos and Maroun (2020), each integrated report is reviewed to identify sections dealing with the organisations’ strategy. The index has a minimum value of 0 (implying low quality) and a maximum value of 1 (indicating high quality). Based on Pitrakkos and Maroun (2020), it is formulated as follows:

5 $${\mathrm{Strategy\, index}}_{it}= \frac{{x}_{i}}{c}$$

where *x*_*i*_ is the strategy disclosure score for firm *i* and *c* is the maximum score assigned (being 3). Where environmental issues (including climate change) do not feature in a firm’s strategy, the strategy disclosure score is 0. Where a firm’s strategy disclosures deal only with environmental issues in general, the strategy disclosure score is 1. If climate change is identified as a strategic issue, but the disclosures are only supported by a high-level or generic explanation of the implications of climate change for the company’s business model, the strategy disclosure score is 2. When climate change is included as part of a proactive approach to reporting on a firm’s overall strategy, the strategy disclosure score is 3 (Pitrakkos and Maroun 2020).

Integration index_*it*_ is constructed and used to measure a multi-dimensional perspective on a firm that recognises how different types of capital interact with an organisation’s strategy, policies, and processes to create value (Pitrakkos and Maroun 2020). According to Pitrakkos and Maroun (2020), this index is the ratio of the number of categories or sections an entity has included in its corporate report to the total number of categories disclosed per disclosure theme (see Table 2). A higher integration index shows that different carbon-related issues are being discussed in multiple parts of an integrated report and suggests higher levels of integration, which, in turn, imply higher quality reporting. On the contrary, a lower integration index suggests that carbon disclosures are limited to specific parts of a corporate report. Based on Pitrakkos and Maroun (2020), it is formulated as follows:

6 $${\mathrm{Integration\, index}}_{it}= \frac{{x}_{i}}{c}$$

where *x*_*i*_ is the total number of categories an entity has disclosed for carbon-related data per the data checklist for a firm *i* and *c* is the total number of categories as per the data checklist (which is 36).

Readability index_*it*_ is constructed and used to measure how easy it is for stakeholders to access and understand the information contained in the report (Pitrakkos and Maroun 2020). According to Pitrakkos and Maroun (2020), the Flesch Reading Ease is used for gauging readability, and it takes into account the number of syllables in the words found in an integrated report as well as sentence length. A higher Flesch Reading Ease score indicates a writing style that is easier to read. The index has a minimum value of 0 (implying low quality—difficult to read) and a maximum value of 1 (indicating high quality—easy to read). Based on Pitrakkos and Maroun (2020), it is formulated as follows:

7 $${\mathrm{Readability\, index}}_{it}= \frac{{G}_{i}}{n}$$

where *G*_*i*_ is the Flesch Reading Ease measure determined by du Toit (2017) for firm *i*. The score for each company is expressed relative to the maximum readability (*n*) score to provide a relative measure of readability.

Assurance index_*it*_ is constructed and used to measure whether carbon disclosures have been independently assured as well as the level of assurance. The index has a minimum value of 0 (implying low quality) and a maximum value of 1 (indicating high quality). Based on Pitrakkos and Maroun (2020), it is formulated as follows:

8 $${\mathrm{Assurance\, index}}_{it}=a$$

where *a* is 0 if no external assurance is provided over an entity’s carbon emissions, 0.5 if limited assurance is provided and 1 if reasonable assurance is provided.

Repetition index_*it*_ is constructed and used to measure how often a specific disclosure is repeated (Pitrakkos and Maroun 2020). The index has a minimum value of 0 (implying high quality—no repetition) and a maximum value of 1 (indicating low quality—significant repetition). Based on Pitrakkos and Maroun (2020), it is formulated as follows:

9 $${\mathrm{Repetition\, index}}_{it}= \frac{1}{{n}_{i}}\sum\nolimits_{j=1}^{{k}_{i}}{{\text{RCD}}}_{ij}$$

where *n*_*i*_ is the total number of carbon disclosures found within the report of firm *i*, RCD_*ij*_ is 1 where disclosure item *j* for firm *i* is registered on the data checklist and the same disclosure was identified previously. RCD_*ij*_ is, otherwise, 0 (Pitrakkos and Maroun 2020).

#### Carbon dioxide emission performance measure

We use the carbon intensity index developed by Hoffman and Busch (2008) as a metric of carbon emission performance (CEP_*it*_) since it is a quantitative and relatively more objective measure (Luo 2019). Generally, Hoffmann and Busch (2008) suggest that carbon emission intensity relates to a firm’s physical carbon performance and describes the extent to which its business activities are based on carbon usage for a defined scope and fiscal year. A higher value of carbon emission intensity suggests that a firm uses its resources, particularly energy, inefficiently and is therefore a poor performer.

Following Hoffmann and Busch (2008) and Luo (2019), we formulate carbon emission performance as the natural logarithm of the ratio of total scope 1,^2^ scope 2^3^ and scope 3^4^ greenhouse gas emissions to the total sales of a firm *i* in year *t*, reflecting the efficiency of its production processes. Therefore, carbon emission performance is calculated as follows:

10 $${{\text{CEP}}}_{it}=\frac{\sum_{k=1}^{{k}_{I}}{C}_{{I}_{kt}}}{{\mathrm{Total\, sales}}_{it}}$$

where the CEP_*it*_ is based on a company’s greenhouse gas emissions, measured in CO_2_ equivalents and denoted by *k* = 1,…, *K*_0_.

#### World economic policy uncertainty measure

In this study, we use the World Uncertainty Index (WUI_*It*_) developed by Ahir et al. (2022) as an uncertainty measure for 143 countries over the period 2005–2020. The economic uncertainty index developed by Ahir et al. (2022) provides major political and economic issues in each country as well as analysis and forecasts on political and economic conditions (2007–2008 global financial crisis, 9/11 attack, Arab Spring, Gulf War II, El Nino crisis, SARS outbreak), which are created by domestic analysts and the editorial board of the Economist. According to Gozgor et al. (2019), WUI_*It*_ is superior to other uncertainty measures since it is the first method to construct an index of uncertainty for a panel dataset of developed and developing countries. In other words, it is comparable across countries. A higher number of WUI_*It*_ means higher uncertainty.

In line with Ahir et al. (2022), Fig. 2 shows that the WUI_*It*_ spikes near the 9/11 attack, SARS outbreak, Gulf War II, Euro debt crisis, El Nino, European border crisis, UK Brexit vote and the 2016 US election. These findings are consistent with previous literature (e.g. Julio and Yook 2012; Da et al. 2014; Baker et al. 2016; Al-Thaqeb and Algharabali 2019), which underline the increase in uncertainty during periods of stock market crashes, world wars, policy-based uncertainty and fiscal crises.

**Fig. 2:**
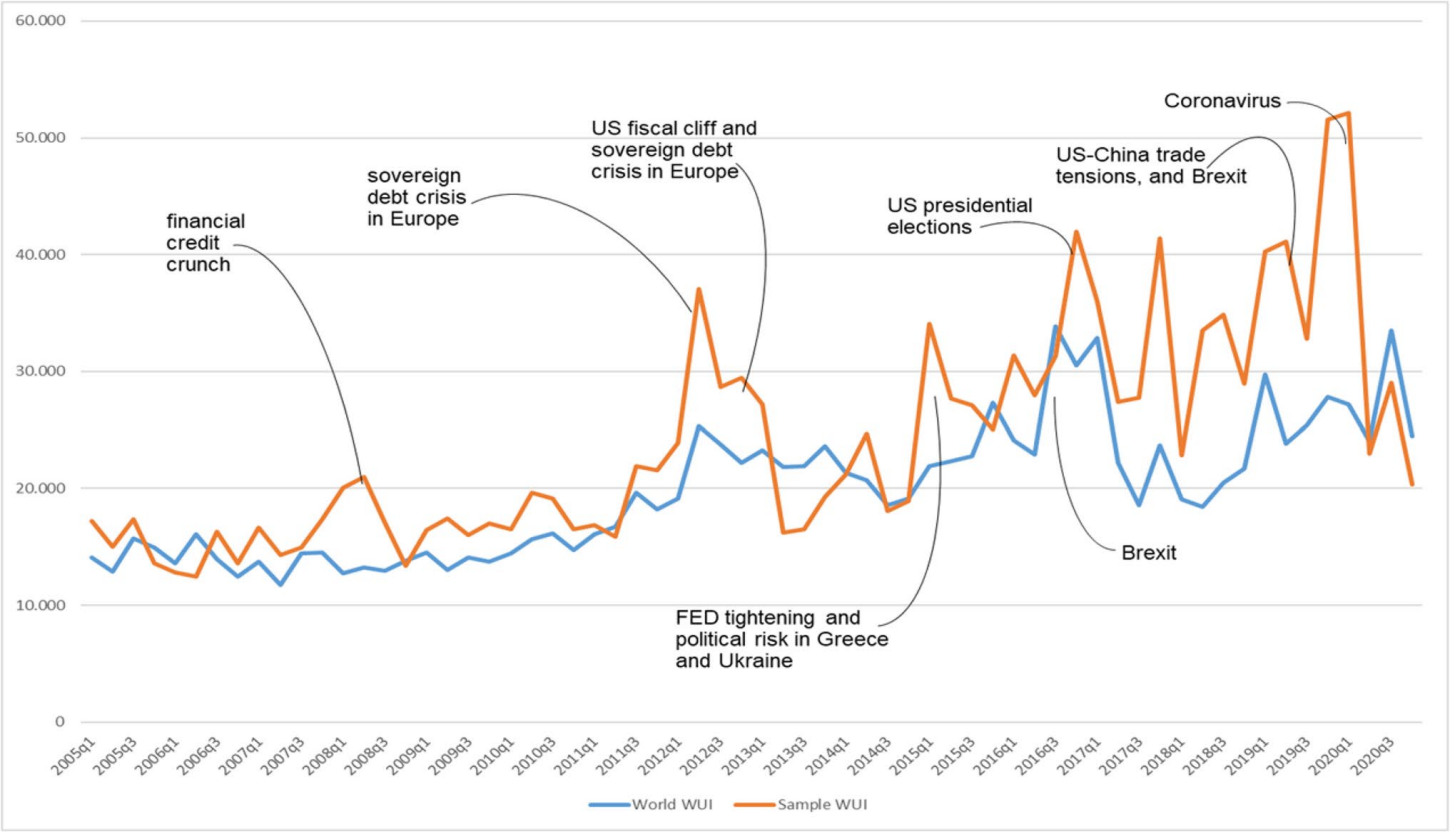
World Uncertainty Index developed by Ahir et al. (2022) (WUIIt)

#### Institutional ownership measure

We use institutional ownership (IO_*it*_) as described by Choi et al. (2012). Therefore, IO_*it*_ refers to stock that is held by investment firms, funds and other large entities rather than individual, retail investors. It is calculated as the proportion of firm shares owned by banks and public pension funds.

#### Industry affiliation measure

Industry affiliation (IA_*it*_) is introduced and constructed by Roberts (1992). He classifies the types of a company industry into two types, namely high-profile industry and low-profile industry. In a low-profile industry, companies are characterised by low consumer and political visibility. Examples of companies that are classified as low-profile include textile and textile products, personal products, household products, building companies, finance and banking, medical equipment suppliers, property, and retail companies. On contrary, in high-profile industry, companies are characterised by high consumer visibility, high political risk, strong level of competition and dangerous or quite risky to health and have a negative impact on society. Examples of companies that are classified as high-profile include automotive, aviation, media and communications, energy (electricity), engineering, health, and transportation and tourism, oil and other mining, chemical, forest, paper, agribusiness, tobacco and cigarettes, food, and beverage products. IA_*it*_ is a dummy variable that takes the value 1 for high-profile industries and 0 otherwise.

### Econometric models

Most of the previous panel analyses have used panel vector autoregressive (PVAR), autoregressive distributed lag (ARDL) and GMM. For the purpose of this research, we use the most implemented model in previous literature (El Ghoul et al. 2018; Gupta 2018; Gozgor et al. 2019), the GMM model developed by Blundell and Bond (1998), because it can be used even when the assumptions of other estimators are not satisfied and it is viewed as a generalisation of many other methods (Radivojević et al. 2019). The GMM model is used to mitigate endogeneity, defined as the casual correlation between the explanatory variable and the error term. Furthermore, following El Ghoul et al. (2018) and Garzón-Jiménez and Zorio-Grima (2021), we lag the dependent variable. Lastly, to provide robustness, we use Hansen’s test and Arellano and Bond’s test to measure if there is over-identification of variables and autocorrelation of errors, respectively.

#### Carbon dioxide emission reporting, institutional ownership and industry affiliation under uncertainty

Using the GMM method, we estimate Eq. (11) to investigate the effects of world economic policy uncertainty, ownership structure and industry affiliation on carbon dioxide emission reporting.

11 $${{\text{CER}}}_{it}= {\beta }_{0}+{\beta }_{1}{{\text{CER}}}_{it-1}+{\beta }_{2}{{\text{WUI}}}_{It}+{\beta }_{3}{{\text{IO}}}_{it}+ {\beta }_{4}{{\text{WUI}}}_{It}\times {{\text{IO}}}_{it}+{\beta }_{5}{{\text{IA}}}_{it}+{\beta }_{6}{{\text{WUI}}}_{It}\times {{\text{IA}}}_{it}+{\beta }_{7}{X}_{it}+{\beta }_{8}{Y}_{It}+\sum\nolimits_{j=1}^{16}{\beta }_{{\text{j}}}\,{{\text{Year}}}_{it}+\sum\nolimits_{j=1}^{4}{\beta }_{j}\,{{\text{Regions}}}_{it}+{\varepsilon }_{it}$$

where CER_*it*_ denotes the current and lagged carbon dioxide emission reporting measured by using quality of carbon dioxide emission reporting developed by Pitrakkos and Maroun (2020) (as presented in section ‘Carbon dioxide emission reporting measure’), WUI_*It*_ is the world economic policy uncertainty developed by Ahir et al. (2022) (as presented in section ‘World economic policy uncertainty measure’), IO_*it*_ is the institutional ownership as described by Choi et al. (2012) (as presented in section ‘Institutional ownership measure’), IA_*it*_ is the industry affiliation developed by Roberts (1992) (as presented in section ‘Industry affiliation measure’), X_*it*_ represents the vector of firm-level control variables and *Y*_*It*_ represents the vector of country-level control variables. Finally, $\sum\nolimits_{j=1}^{16}{\beta }_{j}\,{{\text{Year}}}_{it}$, $\sum\nolimits_{j=1}^{4}{\beta }_{j}\,{{\text{Regions}}}_{it}$ and *ε*_*it*_ denote the ‘time fixed-effects’, the ‘region fixed-effects’ and the ‘error term’, respectively.

Following Galani et al. (2012), Choi et al. (2013), Luo et al. (2012), Hermawan et al. (2018) and Ambarwati and Wicaksono (2020), we include a set of control variables that may affect carbon dioxide emission reporting. These variables include firm size (FIRZ_*it*_), measured as the natural logarithm of total assets; firm profitability (PROR_*it*_), measured as the return on assets; firm leverage (LEVR_*it*_), measured as the ratio of total debt divided by the total assets; firm age (FA_*it*_), measured as the number of years since listing; firm capital expenditure (CAPEX_*it*_), measured as capital expenditures divided by the total sales revenues; Environmental Performance Index (EPI_*It*_) developed by Yale University and Columbia University, which quantifies and numerically benchmarks the environmental performance of a country; the carbon emission trading system (ETS_*It*_) is an indicator that is equal to 1 if the firm is in a country that has an established ETS and 0 otherwise; the Kyoto Protocol (KYOTO_*It*_) is an indicator variable that is equal to 1 if the firm is in a country that has ratified or accepted the Kyoto Protocol and 0 otherwise; and six dimensions of national culture developed by Hofstede (1980, 2001) (power distance (PODI_*It*_),^5^ individualism versus collectivism (INDI_*It*_),^6^ masculinity versus femininity (MAFE_*It*_),^7^ uncertainty avoidance (UNAV_*It*_),^8^ long-term orientation (LOTO_*It*_)^9^ and indulgence versus restraint (INDU_*It*_)^10^).

Luo et al. (2012) state that larger firms disclose more information about carbon emissions from small firms in their reports to gain support from stakeholders and legitimacy from the community. Firm profitability is found to have a positive influence on carbon emission reporting, suggesting that firms with high profitability disclose information that they can act well under environmental pressure (Choi et al. 2013; Hermawan et al. 2018). Kolsi (2017) and Saraswati et al. (2021) suggest that firms with high leverage are at a danger point, which makes firms more conservative in making policies, especially spending. Ambarwati and Wicaksono (2020) reveal that there is a positive association between carbon emission disclosure and firm age, suggesting that older firms have a greater percentage of gaining a good reputation in social responsibility and greater legitimacy than younger firms. Luo and Tang (2014) and Tang and Luo (2016) argue that firms with better environmental performance should report more to show their superior performance. They also illustrate that the culture of a country hardly affects actual environmentally conscious behaviour. Pucheta-Martínez and Gallego-Álvarez (2020) show that firms operating in countries with individualist, masculine and indulgent cultures are less likely to disclose environmental information. Contrary to their predictions, cultures with a long-term orientation also discourage the reporting of environmental information, while uncertainty avoidance contexts tend to promote more environmental reporting. A firm in a country with an ETS or that has accepted/ratified the Kyoto Protocol has more pressure to perform carbon emission reduction activities and therefore a higher level of ecological transparency, which is in favour of stakeholders’ demands (Luo and Tang 2016).

#### Carbon emission dioxide performance, ownership structure and industry affiliation under uncertainty

Using the GMM method, we estimate Eq. (12) to investigate the effects of world economic policy uncertainty, ownership structure and industry affiliation on carbon dioxide emission performance.

12 $${{\text{CEP}}}_{it}= {\beta }_{0}+{\beta }_{1}{{\text{CEP}}}_{it-1}+ {\beta }_{2}{{\text{WUI}}}_{It}+ {\beta }_{3}{{\text{IO}}}_{it}+ {\beta }_{4}{{\text{WUI}}}_{It}\times {{\text{IO}}}_{it}+ {\beta }_{5}{{\text{IA}}}_{it}+ {\beta }_{6}{{\text{WUI}}}_{It}\times {{\text{IA}}}_{it}+ {\beta }_{7}{X}_{it}+ {\beta }_{8}{Y}_{It}+\sum\nolimits_{j=1}^{16}{\beta }_{{\text{j}}}\;{{\text{Year}}}_{it}+\sum\nolimits_{j=1}^{4}{\beta }_{{\text{j}}}\;{{\text{Regions}}}_{it}+ {\upvarepsilon }_{it}$$

where CEP_*it*_ denotes the current and lagged carbon emission performance developed by Hoffman and Busch (2008) (as presented in Eq. (10)), WUI_*It*_ is the world economic policy uncertainty developed by Ahir et al. (2022) (as presented in Eq. (12)), IO_*it*_ is the institutional ownership as described by Choi et al. (2012) (as presented in Eq. (12)), IA_*it*_ is the industry affiliation developed by Roberts (1992) (as presented in Eq. (12)), *X*_*it*_ represents the vector of firm-level control variables and *Y*_*It*_ represents the vector of country-level control variables. Finally, $\sum_{j=1}^{16}{\beta }_{j}\,{{\text{Year}}}_{it}$, $\sum_{j=1}^{4}{\beta }_{j}\,{{\text{Regions}}}_{it}$ and ${\varepsilon }_{it}$ denote the ‘time fixed-effects’, the ‘region fixed-effects’ and the ‘error term’, respectively.

Following Clarkson et al. (2011), Haque (2017) and Khaled (2021), we include a set of control variables that may affect carbon emission performance. These variables include firm size (FIRMZ_*it*_), measured as the natural logarithm of total assets; profitability (PROR_*it*_), measured as the return on assets; firm leverage (LEVR_*it*_), measured as the debt-to-equity ratio; firm liquidity (LIQR_*it*_), measured as the current ratio; employee size (EMPL_*it*_), measured as the natural logarithm of the number of employees; gross domestic product (GDP_*It*_), measured as the natural logarithm of gross domestic product; and worldwide governance indicators, which capture six key dimensions of the quality of governance (voice and accountability (VOAC_*It*_),^11^ political stability and lack of violence (POST_*It*_),^12^ government effectiveness (GOEF_*It*_),^13^ regulatory quality (REQU_*It*_),^14^ rule of law (RULA_*It*_)^15^ and control of corruption (COCO_*It*_)^16^).

Clarkson et al. (2011), Haque (2017) and Khaled (2021) assume that large firms need to maintain their economic scale in terms of products, sales and employees and thus cause greater carbon dioxide emission performance. Furthermore, Clarkson et al. (2011) and Khaled (2021) show that profitability has a significant effect on carbon dioxide emission performance. Haque (2017) finds that firms with a higher number of employees are likely to cause greater carbon dioxide emissions. De Villiers et al. (2011) and Khaled (2021) note that firms with good cash flows are in a better position to invest in substantial environmental projects, allocate funds for compliance costs and improve the environmental situation. Therefore, they claim that liquidity is positively associated with carbon emission performance. Clarkson et al. (2011) and Khaled (2021) show that leverage has a significant effect on carbon emission performance. Hassan and Romilly (2017) note that a higher gross domestic product is associated with higher levels of economic activity and higher greenhouse gas emissions, implying a positive relationship. Halkos and Tzeremes (2013) illustrate that the carbon emission-governance relationship is nonlinear. Gani (2012) and Sarwar and Alsaggaf (2021) indicate that worldwide governance indicators are negatively correlated with carbon dioxide emissions.

The specific descriptions of variables are shown in Table 8 in the Appendix.

## Empirical results

### Descriptive analysis

Table 3 reports the descriptive statistics for the dependent, independent and control variables in our analysis, which are calculated to know the shape of the data under the sample period and include the number of observations, mean and median values of all the variables, and their standard deviations. For example, the mean values of carbon dioxide emission reporting and performance are 4.86 and 0.36, with standard deviations of 0.51 and 0.09, respectively.

**Table 3:** Descriptive statistics

Variables	Description	Observations (no. of firms)	Mean	Standard deviation	Min	Median	Max
CAPEX_*it*_	Firm capital expenditure	604	0.17	0.14	0.02	0.07	0.90
CER_*it*_	Carbon dioxide emission reporting	604	4.86	0.51	3.96	4.36	5.81
CEP_*it*_	Carbon emission performance	604	0.36	0.09	0.35	0.41	0.45
COCO_*It*_	Control of corruption	604	0.55	0.97	− 1.13	0.36	2.07
EMPL_*it*_	Employees size	604	4.82	0.51	2.18	4.81	6.19
EPI_*It*_	Environmental Performance Index	604	80.62	4.74	68.41	79.61	96.74
ETS_*It*_	Carbon emission trading system	604	0.32	0.47	0.00	0.00	1.00
FA_*it*_	Firm age	604	10.00	5.32	1.00	11.00	16.00
FIRZ_*it*_	Firm size	604	5.01	0.15	5.29	5.67	5.84
GDP_*It*_	Gross domestic product	604	12.23	0.43	11.33	12.22	13.33
GOEF_*It*_	Government effectiveness	604	0.74	0.76	− 0.50	0.52	1.90
IC_*it*_	Industry affiliation	604	0.91	0.41	0.00	1.00	1.00
INDI_*It*_	Individualism versus collectivism	604	61.39	28.27	14.00	67.00	91.00
INDU_*It*_	Indulgence versus restraint	604	48.97	18.38	20.00	48.00	97.00
KYOTO_*It*_	Kyoto Protocol	604	0.60	0.51	0.00	1.00	1.00
LEVR_*it*_	Firm leverage	604	0.71	0.10	0.39	0.71	0.99
LIQR_*it*_	Firm liquidity	604	1.41	0.19	1.18	1.47	1.72
LOTO_*It*_	Long-term orientation	604	59.97	27.80	21.00	63.00	100.00
MAFE_*It*_	Masculinity versus femininity	604	65.12	14.95	36.00	62.00	95.00
OS_*it*_	Institutional ownership	604	0.71	0.11	0.62	0.73	0.79
PODI_*It*_	Power distance	604	54.43	17.46	35.00	54.00	95.00
POST_*It*_	Political stability and lack of violence	604	0.05	0.76	− 2.01	0.30	1.27
PROR_*it*_	Firm profitability	604	6.01	3.71	− 1.15	5.01	12.82
REQU_*It*_	Regulatory quality	604	0.71	0.78	− 0.63	0.84	1.93
RULA_*It*_	Rule of law	604	0.61	0.91	− 0.97	0.45	1.92
UNAV_*It*_	Uncertainty avoidance	604	57.55	23.07	30.00	46.00	95.00
VOAC_*It*_	Voice and accountability	604	0.43	0.99	− 1.91	0.75	1.51
WUI_*It*_	World Uncertainty Index	604	24,047.00	8019.80	13,797.32	20,266.95	41,424.80

### Univariate analysis

Tables 4 and 5 present the Pearson correlation matrix for Eqs. (11) and (12). Consistent with our expectations, Table 4 shows that there is a significant positive association between WUI_*It*_ and CER_*it*_ (*p* < 0.05), suggesting that firms increase carbon dioxide emission disclosure in order to minimise the negative effects of uncertainty. The results of Table 4 suggest that the relationship between CER_*it*_ and IC_*it*_ is positive at the 10% significance level, supporting Hypothesis 5. In addition, CER_*it*_ increases with OS_*it*_, suggesting that firms with higher institutional ownership increase carbon dioxide emission reporting. In both cases, WUI_*It*_ moderates the effects of IC_*it*_ and OS_*it*_ on CER_*it*_.

**Table 4:** Pearson correlation coefficients for Eq. (11)

	CER_*it*_	WUI_*It*_	IO_*it*_	WUI_*It*_ × IO_*it*_	IA_*it*_	WUI_*It*_ × IA_*it*_	FIRZ_*it*_	PROR_*it*_	LEVR_*it*_	FA_*it*_	CAPEX_*it*_	EPI_*It*_	ETS_*It*_	KYOTO_*It*_	PODI_*It*_	INDI_*It*_	MAFE_*It*_	UNAV_*It*_	LOTO_*It*_	INDU_*It*_
CER_*it*_	1																			
WUI_*It*_	0.035**	1																		
IO_*it*_	0.282**	0.233	1																	
WUI_*It*_ × IO_*it*_	0.238**	0.609*	0.913**	1																
IA_*it*_	0.262*	− 0.461	0.130**	− 0.085	1															
WUI_*It*_ × IA_*it*_	0.167**	0.707**	0.354*	0.587*	0.301	1														
FIRZ_*it*_	0.358*	− 0.322	0.176*	0.007	− 0.027	− 0.364	1													
PROR_*it*_	0.204	− 0.794**	− 0.167	− 0.457	0.268	0.638**	0.394	1												
LEVR_*it*_	− 0.072**	0.746*	0.137	0.200	− 0.389	0.486*	− 0.739**	− 0.717*	1											
FA_*it*_	0.074**	0.737*	0.251	0.513*	0.005	0.793**	− 0.369**	− 0.827*	0.596*	1										
CAPEX_*it*_	0.143	0.762*	0.259	0.522*	− 0.018*	0.666**	− 0.326*	− 0.861*	0.649*	0.942**	1									
EPI_*It*_	0.619	0.619	0.248	0.418*	0.181*	0.751*	0.518***	0.555	0.544*	0.519*	0.882	1								
ETS_*It*_	0.519**	0.316	0.316	0.316***	0.418*	0.519**	0.357	0.418	− 0.588**	0.448*	0.358	0.505	1							
KYOTO_*It*_	0.018**	0.378	0.518**	0.819**	− 0.619**	0.550**	0.480	− 0.718	− 0.753	0.228*	− 0.618**	0.348	0.550**	1						
PODI_*It*_	0.316	0.215*	0.319**	0.061	0.316	0.418	0.718	− 0.518**	0.951	0.247	0.250	0.559	0.458**	0.418	1					
INDI_*It*_	− 0.518***	0.619*	0.418	0.017	0.510	− 0.718	0.048*	− 0.883*	0.358	− 0.418	0.449**	− 0.458**	− 0.159**	0.519	0.518**	1				
MAFE_*It*_	− 0.234*	− 0.699*	− 0.144	− 0.416	0.123	− 0.650**	0.165	0.617*	− 0.616*	− 0.681**	− 0.691**	0.349**	− 0.348*	0.357	0.817**	− 0.700	1			
UNAV_*It*_	0.369	0.518	0.518*	0.357*	0.347	0.418	0.071	0.999**	0.815**	0.518	0.544	0.340*	0.349	0.518	0.319	− 0.357*	0.430	1		
LOTO_*It*_	0.220**	0.848*	0.285	0.584*	− 0.302	0.666**	− 0.034**	− 0.914*	0.710*	0.922**	0.926**	0.050*	0.478	0.619	− 0.917**	0.228	− 0.731*	0.417**	1	
INDU_*It*_	− 0.218**	− 0.862*	− 0.237	− 0.581*	0.304	− 0.681**	0.315	0.915**	− 0.709**	− 0.915*	− 0.921*	0.232	0.493	0.353	0.719	0.404	0.768*	0.555	− 0.997**	1

Asterisks indicate statistical significance at the 1% (***), 5% (**) and 10% (*) levels, respectively. Variance of inflation factor (VIF) is 3519

**Table 5:** Pearson correlation coefficients for Eq. (12)

	CEP_*it*_	WUI_*It*_	IO_*it*_	WUI_*It*_ × IO_*it*_	IA_*it*_	WUI_*It*_ × IA_*it*_	FIRZ_*it*_	PROR_*it*_	LEVR_*it*_	LIQR_*it*_	EMPL_*it*_	GDP_*It*_	VOAC_*It*_	POST_*It*_	GOEF_*It*_	REQU_*It*_	RULA_*It*_	COCO_*It*_
CEP_*it*_	1																	
WUI_*It*_	− 0.933***	1																
IO_*it*_	− 0.224	0.234	1															
WUI_*It*_ × IO_*it*_	− 0.574	0.609*	0.913**	1														
IA_*it*_	− 0.310**	− 0.460**	0.130	− 0.085	1													
WUI_*It*_ × IA_*it*_	− 0.752*	0.707*	0.350	0.587**	0.300	1												
FIRZ_*it*_	0.351**	− 0.320	0.018	0.007	− 0.027	− 0.364	1											
PROR_*it*_	0.887***	0.794**	− 0.208	− 0.457	0.273	− 0.638**	0.394	1										
LEVR_*it*_	− 0.751***	0.746*	− 0.189	0.200	− 0.389	0.486	− 0.739***	− 0.717**	1									
LIQR_*it*_	0.666**	0.666**	0.040	− 0.246	0.431	− 0.364	0.593*	0.631*	− 0.912**	1								
EMPL_*it*_	0.872	0.704**	0.018	0.303	− 0.197	0.596*	− 0.169	− 0.775*	0.587**	− 0.474	1							
GDP_*It*_	0.929**	0.762**	0.295	0.522*	− 0.185	0.666**	− 0.328	− 0.861*	0.649*	− 0.580**	0.874**	1						
VOAC_*It*_	− 0.818	0.766**	0.037	− 0.295*	0.588**	− 0.351	0.062	0.749**	− 0.640**	− 0.624***	− 0.855**	− 0.725**	1					
POST_*It*_	− 0.228	− 0.460	− 0.029	− 0.535**	0.389	− 0.182	0.117	0.115	− 0.313	0.270**	0.404	0.088	0.219**	1				
GOEF_*It*_	− 0.518	0.605	0.036**	− 0.316	0.819	− 0.186	0.318*	0.618**	0.588	0.618	0.519	0.455	0.351	0.442	1			
REQU_*It*_	− 0.318	0.518	0.347**	− 0.418	0.517	− 0.359	0.619**	0.333*	0.667	0.480**	0.347	0.555	0.254	0.521	0.418*	1		
RULA_*It*_	− 0.413	0.176	0.356**	0.363	0.619	0.647**	− 0.227*	− 0.430	0.156*	− 0.117*	0.509**	0.534*	− 0.311**	0.285	0.517**	0.419	1	
COCO_*It*_	− 0.375	0.317	0.147*	0.718	0.316	0.418*	0.915	− 0.518	0.669	− 0.715**	0.613	0.351	0.810	0.321	0.315*	0.357	0.458	1

Asterisks indicate statistical significance at the 1% (***), 5% (**) and 10% (*) levels, respectively. Variance of inflation factor (VIF) is 5916

Table 5 illustrates that the significant coefficient between WUI_*It*_ and CEP_*it*_ (Pearson =  − 0.933***) provides support for our expectation that firms increase carbon dioxide emissions during periods of uncertainty. In addition, despite the predictions that OS_*it*_ would have a negative impact on CEP_*it*_, we find a non-significant correlation. Our results indicate a negative relationship between IC_*it*_ and CER_*it*_ with a significance level of 5%. Finally, the Pearson correlation matrix in Table 5 provides evidence that WUI_*It*_ moderates the effect of IC_*it*_ on CER_*it*_.

In sum, Table 4 and 5 show that the variance inflation factors (VIFs) for both models are well below 10, as in Griffin et al. (2017) and Shad et al. (2020), thus indicating no evidence of a multicollinearity problem.

### Multivariate analysis

#### Carbon dioxide emission reporting, institutional ownership and industry affiliation under uncertainty

First, in panel A of Table 6, we report that the WUI_*It*_ coefficient is significant and has a positive value, which is also ratified in panels B and C (when the other hypotheses are also tested). Consistent with Hypothesis 1, we find that carbon dioxide emission reporting increases during periods of uncertainty in order to reduce the transparency of carbon dioxide emission information disclosure and lower information asymmetry when the uncertainty is at a high level. Our result is in line with El Ghoul et al. (2018).

**Table 6:** Results of the generalised method of moments (GMM) model (Eq. (11))

Dependent variable	CER_*it*_		
	Panel A	Panel B	Panel C
CER_*it* − 1_	0.963** (0.759)	0.814** (0.345)	0.419** (0.550)
WUI_*It*_	0.357*** (1.872)	1.857*** (1.963)	1.101*** (1.525)
IO_*it*_		99.527** (44.758)	81.752** (31.789)
WUI_*It*_ × IO_*it*_		29.768*** (31.527)	21.528* (33.855)
IA_*it*_			157.852** (99.751)
WUI_*It*_ × IA_*it*_			45.789** (51.475)
FIRZ_*it*_	13.718** (11.822)	16.827*** (3.527)	2.718 (5.628)
PROR_*it*_	0.741 (0.089)	0.096 (0.033)	0.475 (0.042)
LEVR_*it*_	− 7.510*** (11.527)	− 15.741 (5.748)	− 3.222*** (12.417)
FA_*it*_	0.001 (0.001)	0.007** (0.041)	0.052 (0.037)
CAPEX_*it*_	8.572 (3.573)	7.518 (3.555)	9.785 (2.202)
EPI_*It*_	0.228 (2.663)	0.667 (2.448)	0.798 (1.993)
ETS_*It*_	8.615** (3.657)	7.158** (2.418)	7.596* (2.990)
KYOTO_*It*_	0.667** (0.010)	0.758 (0.099)	0.880 (0.128)
PODI_*It*_	9.753 (2.418)	8.157 (3.518)	9.982 (2.555)
INDI_*It*_	− 3.518 (0.518)	− 5.418** (0.357)	− 4.316** (0.660)
MAFE_*It*_	− 4.751*** (2.951)	− 3.418 (1.753)	− 3.444*** (1.555)
UNAV_*It*_	7.519 (1.519)	8.015 (1.637)	6.751 (1.808)
LOTO_*It*_	4.521 (3.758)	3.568** (2.744)	4.752 (1.951)
INDU_*It*_	− 5.718 (5.896)	− 6.798*** (4.857)	− 5.662 (5.440)
Year	Controlled	Controlled	Controlled
Region	Controlled	Controlled	Controlled
Hansen’s test (prob > chi^2^)	0.118	0.348	0.227
Arellano and Bond test (prob > *Z*)	0.375	0.485	0.421
*R*^2^	0.035	0.076	0.011
*N*	5157	5157	5157

Asterisks indicate statistical significance at 1% (***), 5% (**) and 10% (*) levels, respectively, and the standard errors may be found in the parentheses

Second, panel B in Table 6 shows that higher IO_*it*_ increases CER_*it*_, which is also confirmed by panel C. Thus, we have no evidence for rejecting Hypothesis 3, contrary to the findings obtained by Akhiroh and Kiswanto (2016) and Hermawan et al. (2018). This evidence corroborates that firms with greater institutional ownership should disclose more environmental information in order to strengthen their investment confidence and improve shareholders’ interests.

Third, concerning the mediating role of institutional ownership on the carbon dioxide emission reporting-world economic policy uncertainty nexus, panel B of Table 6 indicates that IO_*it*_ × WUI_*It*_ is positive and significant in relation to CER_*it*_, which confirms Hypothesis 3a. These results are in line with the findings in panel C, suggesting that the impact of institutional ownership on carbon dioxide emission reporting is more prominent during periods of high uncertainty.

Fourth, panel C in Table 6 reports that IA_*it*_ is positively associated with CER_*it*_. Thus, consistent with Hypothesis 4, our results indicate that high-profile industries present high carbon dioxide emission reporting, arriving at consistent findings with Matsumura et al. (2014) and Saraswati et al. (2021). Our findings indicate that firms belonging to a high-profile industry characterised by high consumer visibility, high political risk, a strong level of competition and being dangerous or quite risky to health are more likely to disclose more environmental information due to reacting to social and political pressure and maintaining their legitimacy.

Fifth, in panel C of Table 6, we test Hypothesis 4a and incorporate the interaction term between industry affiliation and the World Uncertainty Index (WUI_*It*_ × IA_*it*_) on carbon dioxide emission reporting (CER_*it*_). We indicate that WUI_*It*_ × IA_*it*_ is positive and significant in relation to CER_*it*_. Furthermore, comparing the coefficients on the interaction term with those of IC_*It*_, the impact of industry classification on the carbon dioxide emission reporting is stronger during periods of uncertainty. Such evidence is supportive of our Hypothesis 4a.

Concerning control variables in Table 6, our results show consistency with previous studies (e.g. Luo et al. 2012; Luo and Tang 2016; Tang and Luo 2016; Ambarwati and Wicaksono 2020; Pucheta-Martínez and Gallego-Álvarez 2020; Saraswati et al. 2021). Specifically, Table 6 indicates that larger and older firms disclose more carbon dioxide emission information to gain support from stakeholders and legitimacy from the community. On the contrary, more leveraged firms display lower carbon dioxide emission reporting. Furthermore, Table 6 shows that higher levels of masculinity and indulgence are associated with a lower level of carbon dioxide emission reporting, implying a negative relationship. In the same sense, carbon dioxide emission reporting is higher with a greater long-term orientation. Finally, Table 6 illustrates that firms in countries with an ETS or/and accepted/ratified the Kyoto Protocol have more pressure to disclose more information about carbon dioxide emissions.

Finally, we implement Hansen’s test in Table 6. The results show that we do not reject hypotheses of over-identifying restriction, and the instruments are valid. In addition, using the Arellano and Bond test, Table 6 illustrates that the hypotheses of no serial correlation of errors in Eq. (11) are accepted, taking into account that the probability of the *Z* value is greater than 0.05.

#### Carbon dioxide emission performance, institutional ownership and industry affiliation under uncertainty

First, panel A in Table 7 indicates a negative relationship between CER_*it*_ and WUI_*It*_, which is also confirmed by panels B and C. Therefore, regarding Hypothesis 2, we can conclude that firms decrease energy consumption during periods of uncertainty, which, in turn, has a negative influence on carbon dioxide emission intensity. Our result is in line with Zhou et al. (2018) and Wei et al. (2022) and inconsistent with the findings of Adedoyin and Zakari (2020) and Huang et al. (2020). Thus, Hypothesis 2 is accepted.

**Table 7:** Results of the generalised method of moments (GMM) model (Eq. (12))

Dependent variable	CEP_*it*_		
	Panel A	Panel B	Panel C
CEP_*it* − 1_	0.519** (0.637)	0.617** (0.753)	0.357** (0.457)
WUI_*It*_	− 10.523*** (3.852)	− 15.819*** (5.714)	− 14.527*** (4.753)
IO_*it*_		114.759 (15.579)	146.851 (16.674)
WUI_*It*_ × IO_*it*_		57.819 (10.796)	51.427 (35.415)
IA_*it*_			− 79.815*** (19.618)
WUI_*It*_ × IA_*it*_			− 91.752** (55.741)
FIRZ_*it*_	45.632*** (11.527)	49.631*** (15.275)	40.157 (21.520)
PROR_*it*_	0.325 (0.517)	0.415** (0.359)	0.617*** (0.637)
LEVR_*it*_	− 31.635** (3.528)	− 20.518 (5.628)	− 25.718** (8.762)
LIQR_*it*_	5.417** (6.002)	4.719 (5.410)	7.433 (6.852)
EMPL_*it*_	3.517 (0.859)	4.714 (0.654)	3.555 (0.639)
GDP_*It*_	50.637*** (25.741)	49.637** (21.417)	55.718** (29.555)
VOAC_*It*_	− 25.618 (3.519)	− 19.638 (3.851)	− 12.637 (2.444)
POST_*It*_	− 31.524 (1.519)	− 35.614 (1.951)	− 33.745 (2.410)
GOEF_*It*_	− 16.521 (3.515)	− 21.418 (4.777)	− 19.636 (5.555)
REQU_*It*_	− 19.519 (21.555)	− 11.215 (25.880)	− 15.515 (29.518)
RULA_*It*_	− 22.520 (2.220)	− 29.518 (2.365)	− 30.518 (1.991)
COCO_*It*_	− 51.534 (36.369)	− 36.583 (32.002)	− 39.500 (41.410)
Year	Controlled	Controlled	Controlled
Region	Controlled	Controlled	Controlled
Hansen’s test (prob > chi^2^)	0.223	0.247	0.289
Arellano and Bond test (prob > *Z*)	0.482	0.437	0.493
*R*^2^	0.934	0.835	0.955
*N*	5157	5157	5157

Asterisks indicate statistical significance at 1% (***), 5% (**) and 10% (*) levels, respectively, and the standard errors may be found in the parentheses

Second, panel B of Table 7 shows a negative and insignificant relation between IO_*it*_ and CEP_*it*_, which is also indicated in panel C. Consequently, inconsistent with Liu et al. (2019), we reject Hypothesis 4, suggesting that institutional ownership has no effect on carbon dioxide emission performance.

Third, concerning the effect of the interaction term WUI_*It*_ × IO_*it*_ on CEP_*it*_, panel B in Table 7 shows a positive and insignificant relationship. In this regard, our results indicate that institutional ownership does not play a mediating role in the world economic policy uncertainty-carbon dioxide emission performance nexus, and thus, we reject Hypothesis 4a.

Fourth, consistent with Hypothesis 6, panel C in Table 7 reports that IA_*It*_ is negatively associated with CEP_*it*_. These results are in agreement with the findings of Tian et al. (2014) and Dong et al. (2018) that high-profile industries accelerate carbon emission and carbon dioxide emission performance. This evidence corroborates the sense that since high-profile industries suffer from strong social and political pressures, investors increase their pressure on these firms to incorporate social and environmental considerations into their business activities in order to decrease their carbon dioxide emission footprint.

Fifth, panel C in Table 7 shows the interaction effect of industry affiliation and the World Uncertainty Index (WUI_*It*_ × IA_*It*_) on carbon dioxide emission performance (CEP_*it*_). In this regard, our results confirm the findings of Yuan et al. (2022) regarding the fact that firms in more sensitive industries tend to adopt more corporate social responsibility engagement during periods of higher uncertainty, as it is a positive signal to their stakeholders. Hence, we accept Hypothesis 6a.

Concerning control variables in Table 7, our results show consistency with previous studies (e.g. 154, 158 and 162). Specifically, Table 7 indicates that carbon dioxide emission performance is higher when firm size, profitability and liquidity are higher. On the contrary, we find that firm leverage is negatively associated with carbon dioxide emission performance. Furthermore, our results illustrate those higher levels of economic activity increase energy consumption, which, in turn, accelerates carbon dioxide emission performance.

Finally, Hansen’s test, Arellano test and Bond test are implemented to examine the endogeneity problem of Eq. (12). The results of Table 7 indicate that the hypotheses of over-identifying restriction are not rejected and the instruments are valid. In addition, we accept the hypotheses of no serial correlation of errors in Eq. (12), taking into account that the probability of the *Z* value is greater than 0.05.

## Robustness tests

In the first robustness test, we use the Carbon Disclosure Project (CDP), developed by Choi et al. (2013) (see Table 9 in the Appendix), as an additional proxy for a firm’s carbon emission reporting. This index consists of five categories related to carbon dioxide emissions and climate change, namely climate change (risks and opportunities), greenhouse gas emissions, energy consumption, reduction of greenhouse gases and costs, and accountability for carbon emissions. In these five categories, 18 checklist items are identified. The maximum score is 18 (high carbon dioxide emission reporting), while the minimum score is 0 (low carbon dioxide emission reporting). The results (not shown) are virtually the same in the main test, suggesting that the finding is not sensitive to model specifications.

Next, we use the economic policy uncertainty developed by Baker et al. (2016) for each of the examining countries as an additional measure of uncertainty. The results (not shown) show that the coefficients of the variable are significant and positive, suggesting that carbon dioxide emission reporting increases during periods of high economic policy uncertainty. On the contrary, we find (results not shown) that there is a negative association between economic policy uncertainty and carbon dioxide emission performance. All of the other results are the same as in the main tests, which further corroborates our interpretations.

Finally, we use state ownership as an additional measure of ownership structure. Based on Zeng (2010), we measure state ownership as the percentage of shares owned by shares or state legal persons over the total number of shares. The results (not shown) are consistent with our main theoretical predictions, suggesting that state ownership is an important dimension of corporate governance that has a prominent effect on carbon dioxide emission reporting and performance, decreasing the carbon dioxide emission footprint relative to all other ownership types, even though the state is more likely to retain ownership in high-polluting industries.

## Conclusions and policy implications

Our study explores how the World Economic Policy Uncertainty Index developed by Ahir et al. (2022) impacts carbon dioxide emission reporting and performance by examining a comprehensive set of the Fortune Global 500 list for the fiscal years 2005–2020. We demonstrate that carbon dioxide emission reporting increases when world economic policy uncertainty increases in order to mitigate the negative effects of uncertainty and increase firm trust (El Ghoul et al. 2018). Furthermore, in line with the findings of Saraswati et al. (2021), the results show that firms with greater institutional ownership and firms obtained in high-profile industries have incentives to disclose more carbon dioxide emission information in order to strengthen investors’ confidence and trust, especially during periods of high world economic policy uncertainty.

Similarly, we observe that firms decrease carbon dioxide emission performance during periods of high world economic policy uncertainty (Wei et al. 2022). In addition, we demonstrate that firms belonging to high-profile industries lower their carbon dioxide emission performance due to political and social pressures on these firms to incorporate environmental considerations (Dong et al. 2018), especially during periods of high world economic policy uncertainty (Yuan et al. 2022).

The implications of the study on the relationship between the World Economic Policy Uncertainty Index and carbon dioxide emission reporting and performance have several practical implications across different sectors and areas. First, in times of heightened economic policy uncertainty, it is imperative for firms to modify their strategies pertaining to the reporting of carbon dioxide emissions. According to the findings of the study, increased levels of uncertainty prompt companies to engage in greater disclosure of emission information as a means to alleviate adverse consequences and foster trust. Second, it is essential for companies, particularly those with significant institutional ownership, to utilise enhanced transparency regarding carbon dioxide emissions as a means to bolster investor confidence in times characterised by increased economic policy uncertainty. Third, in times of economic uncertainty, it is important for high-profile industries to disclose comprehensive data regarding carbon emissions in order to maintain investor confidence and trust. Fourth, it is essential for firms to acknowledge that in periods of heightened uncertainty, there may be a tendency to reduce their carbon dioxide emission performance. This underscores the necessity of actively overseeing and upholding rigorous environmental regulations, even in periods of economic instability. Fifth, high-profile industries may encounter increased social and political pressure to reduce emissions, and they may encounter greater difficulties in achieving environmental performance objectives during periods of economic uncertainty. Therefore, it is important for them to formulate effective strategies in order to effectively manage and reconcile these competing demands. Sixth, in light of increased economic uncertainty, it is essential for governments and regulatory bodies to contemplate the modification of policies. It is essential for policies to provide backing to environmental initiatives and foster corporate responsibility, particularly for high-profile industries that are confronted with mounting social and political pressures. Seventh, it is recommended for firms to incorporate economic policy uncertainty as a variable in their evaluations of risk management, with a specific focus on environmental performance and reporting. It is suggested that appropriate measures be taken to formulate contingency plans aimed at mitigating the impact of economic uncertainty on environmental performance fluctuations.

The study makes the following contributions. The primary contribution of our research lies in providing valuable insights into specific domains within the field of environmental accounting and finance. (a) This study examines the fluctuations in carbon dioxide emission reporting and performance during periods of policy-induced uncertainty. (b) Additionally, it investigates the potential impact of institutional ownership and industry affiliation as factors that may mitigate the effects of global economic policy uncertainty on carbon dioxide emission reporting and performance. Our study builds upon existing literature (e.g. Huang et al. 2020; Pirgaip and Dincergok 2020; Adedoyin and Zakari 2020; Yu et al. 2021; Wei et al. 2022) that primarily investigates the impact of economic-induced policy uncertainty on carbon dioxide emission performance. We contribute to this body of research by demonstrating that heightened levels of uncertainty are associated with decreased carbon dioxide emission performance and increased carbon dioxide emission reporting. Furthermore, our study builds upon the research conducted by Yu et al. (2021) and Solikhah (2016) by providing additional evidence that higher levels of institutional ownership are associated with increased disclosure of carbon dioxide emissions and a reduction in carbon dioxide emission performance, particularly in situations characterised by high levels of uncertainty. Similarly, we enhance the findings of prior studies (e.g. Liu et al. 2015; Dong et al. 2018; Faisal et al. 2018; Saraswati et al. 2021) that demonstrate a positive association between prominent industries and the disclosure of carbon dioxide emission information, as well as a negative relationship between these industries and carbon dioxide emission performance. Moreover, we find that these linkages are more pronounced during periods of increased uncertainty. Furthermore, our study contributes to the existing body of literature by conducting an analysis over a span of 16 years, specifically from 2005 to 2020, and utilising a substantial international sample derived from the Fortune Global 500 companies across the globe. Furthermore, we improve the robustness of our findings by incorporating various metrics for carbon emission reporting, economic policy uncertainty and ownership structure.

Finally, we note the following limitations in our research design that could potentially impact our results. Specifically, we exclude the finance and banking industries from our sample since they have different regulations than non-finance industries. Recent papers (e.g. Biswas 2011; Bahl 2012; Meena 2013; Nath et al. 2014; Lalon 2015) focus on the analysis of green banking as a new strategic imperative and as an initiative for sustainable development. Therefore, future research shall analyse the carbon dioxide emission reporting and performance of the finance and banking industries and juxtapose these results with the findings of this research. In addition, it is recommended to undertake a more targeted longitudinal investigation of particular industries or regions in order to gain a comprehensive understanding of the influence of economic policy uncertainty on carbon dioxide emissions. This may entail conducting a comprehensive analysis of specific industries, such as energy, manufacturing or technology, in order to comprehend the impact of policy modifications on emission reporting and performance trends. Furthermore, future research should investigate the potential effects of technological innovations and advancements on the mitigation of carbon emissions in the context of economic uncertainty. This may entail an examination of how companies utilise technology or innovation to sustain emission reduction strategies in the face of economic instability.

## Data Availability

The datasets used and/or analysed during the current study are available from the corresponding author on reasonable request.

## Appendix

**Table 8:** Variable definitions

Variable	Definitions
CAPEX_*it*_	Firm capital expenditure measured as capital expenditures divided by the total sales revenues
CEP_*it*_	Carbon emission performance developed by Hoffman and Busch (2008)
CER_*it*_	Carbon dioxide emission reporting using quality of carbon dioxide reporting index developed by Pitrakkos and Maroun (2020)
COCO_*It*_	Control of corruption is a key dimension of the quality of governance developed by Worldwide Governance Indicators
EMPL_*it*_	Employee’s size measured as the natural logarithm of the number of employees of the firm
EPI_*It*_	Environmental Performance Index developed by Yale University and Columbia University which quantifies and numerically benchmarks the environmental performance of a country
ETS_*It*_	Carbon emission trading system is an indicator that is equal to 1 if the firm is in country that has an established ETS and 0 otherwise
FA_*it*_	Firm age measured as the number of years since listing
FIRZ_*it*_	Firm size measured as the natural logarithm of total assets
GDP_*It*_	Gross domestic product measured as the natural logarithm of gross domestic product
GOEF_*It*_	Government effectiveness is a key dimension of the quality of governance developed by Worldwide Governance Indicators
IA_*it*_	Industry affiliation developed by Roberts (1992)
INDI_*It*_	Individualism as a dimension of national culture developed by Hofstede (1980, 2001)
INDU_*It*_	Indulgence as a dimension of national culture developed by Hofstede (1980, 2001)
IO_*it*_	Institutional ownership as described by Choi et al. (2012)
KYOTO_*It*_	Kyoto Protocol is an indicator variable that is equal to 1 if the firm is in a country that has ratified or accepted the Kyoto Protocol and 0 otherwise
LEVR_*it*_	Leverage ratio measured as the debt-to-equity ratio
LIQR_*it*_	Liquidity ratio measured as the current ratio
LOTO_*It*_	Long-term orientation as a dimension of national culture developed by Hofstede (1980, 2001)
MAFE_*It*_	Masculinity as a dimension of national culture developed by Hofstede (1980, 2001)
PODI_*It*_	Power distance as a dimension of national culture developed by Hofstede (1980, 2001)
POST_*It*_	Political stability and lack of violence is a key dimension of the quality of governance developed by Worldwide Governance Indicators
PROR_*it*_	Firm profitability ratio using the return on assets
REQU_*It*_	Regulatory quality is a key dimension of the quality of governance developed by Worldwide Governance Indicators
RULA_*It*_	Rule of law is a key dimension of the quality of governance developed by Worldwide Governance Indicators
UNAV_*It*_	Uncertainty avoidance as a dimension of national culture developed by Hofstede (1980, 2001)
VOAC_*It*_	Voice and accountability is a key dimension of the quality of governance developed by Worldwide Governance Indicators
WUI_*It*_	World Economic Policy Uncertainty Index developed by Ahir et al. (2022)

**Table 9:** Carbon emission disclosure checklist

Category	Items
1. Climate change: risks and opportunities (2 items)	CC1, assessment/description of the risks (regulatory, physical or general) relating to climate change and actions taken or to be taken to manage the risks
	CC2, assessment/description of current (and future) financial implications, business implications and opportunities of climate change
2. GHG emissions accounting (7 items)	GHG1, description of the methodology used to calculate GHG emissions (e.g. GHG protocol or ISO)
	GHG2, existence external verification of quantity of GHG emission; if so, by whom and on what basis
	GHG3, total GHG emissions; metric tons CO_2_-e emitted
	GHG4, disclosure of scopes 1 and 2 or scope 3 direct GHG emissions
	GHG5, disclosure of GHG emissions by sources (e.g. coal, electricity)
	GHG6, disclosure of GHG emissions by facility or segment level
	GHG7, comparison of GHG emissions with previous years
3. Energy consumption accounting (3 items)	EC1, total energy consumed (e.g. terajoules or petajoules)
	EC2, quantification of energy used from renewable sources
	EC3, disclosure by type, facility or segment
4. GHG reduction and cost (4 items)	RC1, detail of plans or strategies to reduce GHG emissions
	RC2, specification of GHG emission reduction target level and target year
	RC3, emission reductions and associated costs or savings achieved to date as a result of the reduction plan
	RC4, cost of future emissions factored into capital expenditure planning
5. Carbon emission accountability (2 items)	ACC1, indication of which board committee (or other executive body) has overall responsibility for actions related to climate change
	ACC2, description of the mechanism by which the board (or other executive body) reviews the company’s progress regarding climate change

Choi et al. (2013)

## Footnotes

1 According to Roberts (1992) and Hackston and Milne (1996), high-profile industries are characterised by high sensitivity to the environmental and social environment, high consumer visibility, a high level of political risk, or concentrated, intense competition. Furthermore, according to Roberts (1992), low-profile industries are those that do not get much public attention if a company makes a failure or mistake in some aspect of their production process or products.

2 Scope 1 emissions are direct greenhouse emissions that occur from sources that are controlled or owned by an organisation (e.g. emissions associated with fuel combustion in boilers, furnaces and vehicles).

3 Scope 2 emissions are indirect greenhouse gas emissions associated with the purchase of electricity, steam, heat or cooling. Although scope 2 emissions physically occur at the facility where they are generated, they are accounted for in an organisation’s greenhouse gas inventory because they are a result of the organisation’s energy use.

4 Scope 3 emissions are the result of activities from assets not owned or controlled by the reporting organisation, but that the organisation indirectly impacts its value chain. Scope 3 emissions include all sources not within an organisation’s scope 1 and 2 boundary.

5 Power distance ranges from 0 to 100. Scores close to 0 represent a smaller power distance, while scores close to 100 represent a larger power distance.

6 Individualism versus collectivism ranges from 0 to 100. Scores close to 0 represent societies that tend towards collectivism, while scores close to 100 represent individualist societies.

7 Masculinity versus femininity ranges from 0 to 100. Scores close to 0 represent more feminine societies, while scores close to 100 represent more masculine societies.

8 Uncertainty avoidance ranges from 0 to 100. Scores close to 0 represent societies with weaker uncertainty avoidance, while scores close to 100 represent societies with stronger uncertainty avoidance.

9 Long-term orientation ranges from 0 to 100. Scores close to 0 represent a shorter-term orientation, while scores close to 100 represent a longer-term orientation.

10 Indulgence versus restraint ranges from 0 to 100. Scores close to 0 represent a more restrained society, while scores close to 100 represent a more indulgent society.

11 Voice and accountability capture perceptions of the extent to which a country’s citizens are able to participate in selecting their government, as well as freedom of expression, freedom of association and a free media. It ranges from − 2.5 (weak) to 2.5 (strong).

12 Political stability and lack of violence capture perceptions of the likelihood that the government will be destabilised or overthrown by unconstitutional or violent means, including politically motivated violence and terrorism. It ranges from − 2.5 (weak) to 2.5 (strong).

13 Government effectiveness captures perceptions of the quality of public services, the quality of the civil service and the degree of its independence from political pressures, the quality of policy formulation and implementation, and the credibility of the government’s commitment to such policies. It ranges from − 2.5 (weak) to 2.5 (strong).

14 Regulatory quality captures perceptions of the ability of the government to formulate and implement sound policies and regulations that permit and promote private sector development. It ranges from − 2.5 (weak) to 2.5 (strong).

15 Rule of law captures perceptions of the extent to which agents have confidence in and abide by the rules of society, and in particular, the quality of contract enforcement, property rights, the police and the courts, as well as the likelihood of crime and violence. It ranges from − 2.5 (weak) to 2.5 (strong).

16 Control of corruption captures perceptions of the extent to which public power is exercised for private gain, including both petty and grand forms of corruption, as well as ‘capture’ of the state by elites and private interests. It ranges from − 2.5 (weak) to 2.5 (strong).
